# Assessing the impact of oar blade angle on lower back muscle activation during on-water rowing, a pilot study

**DOI:** 10.3389/fspor.2025.1708377

**Published:** 2026-01-09

**Authors:** B. van Trigt, V. G. T. Luidens, S. Bozaci, T. J. A. Luiten, M. van der Laan, A. J. Greidanus

**Affiliations:** 1 Department of Biomechanical Engineering, Delft University of Technology, Delft, Netherlands; 2 Ridgeline Movement, Amsterdam, Netherlands; 3 Laboratory for Aero & Hydrodynamics, Delft University of Technology, Delft, Netherlands

**Keywords:** electromyography, erector spinae, low back pain, injuries, biomechanics, sports

## Abstract

**Background:**

Rowing is a sport that places significant stress on the lower back, often leading to low back pain (LBP) injuries among athletes. Laboratory studies have shown that rowing with an oar blade under an angle is more efficient compared to a traditional blade. The effect of blade angle on the lower back is unknown. Therefore, the aim of this study is to investigate the effect of different oar blade angles on the muscle activation of the lower back muscles during on-water rowing.

**Methods:**

Seven collegiate (five males, two females) athletes row 500 m on water twice, once with a traditional (0-degrees blade) and once with an oar blade under a 5-degrees angle. Surface electromyography of the longissimus muscle of the erector spinae was measured bilaterally at the thoracic and lumbar level with a sample frequency of 2,000 Hz. In total 1,443 strokes were analyzed. Statistical Parametric Mapping was used to investigate the differences in muscle activity between the 0-degrees and 5-degrees oar blade.

**Results:**

No significant differences in muscle activity were found between the 0- and 5-degrees oar blade.

**Conclusion:**

Rowing with an oar blade under 5-degrees did not alter the muscle activity during on-water rowing. This indicates that rowing with an oar blade under 5-degrees may not increase the muscle activation. These results are important as it seems that a change in oar blade angle does not increase the injury risk, longitudinal studies should investigate the effect of oar blade angles on LBP injuries.

## Introduction

Innovation in rowing has been driven by the desire for faster times, leading to the introduction of various advancements such as sliding seats and lightweight constructions (1). Other innovations are based on the rowing oar, the link between the rower's power output and the water, plays a crucial role in enhancing performance among these technological developments. The oar is built up by the handle, shaft and blade. Over the years new blades have been developed where researchers and designers have explored various designs to optimize rowing efficiency, from the classic Macon blade via the Big Blade to the more recent Comp blades. One of the latest areas of interests is the modification in the oar blade angle.

**Figure 1:**
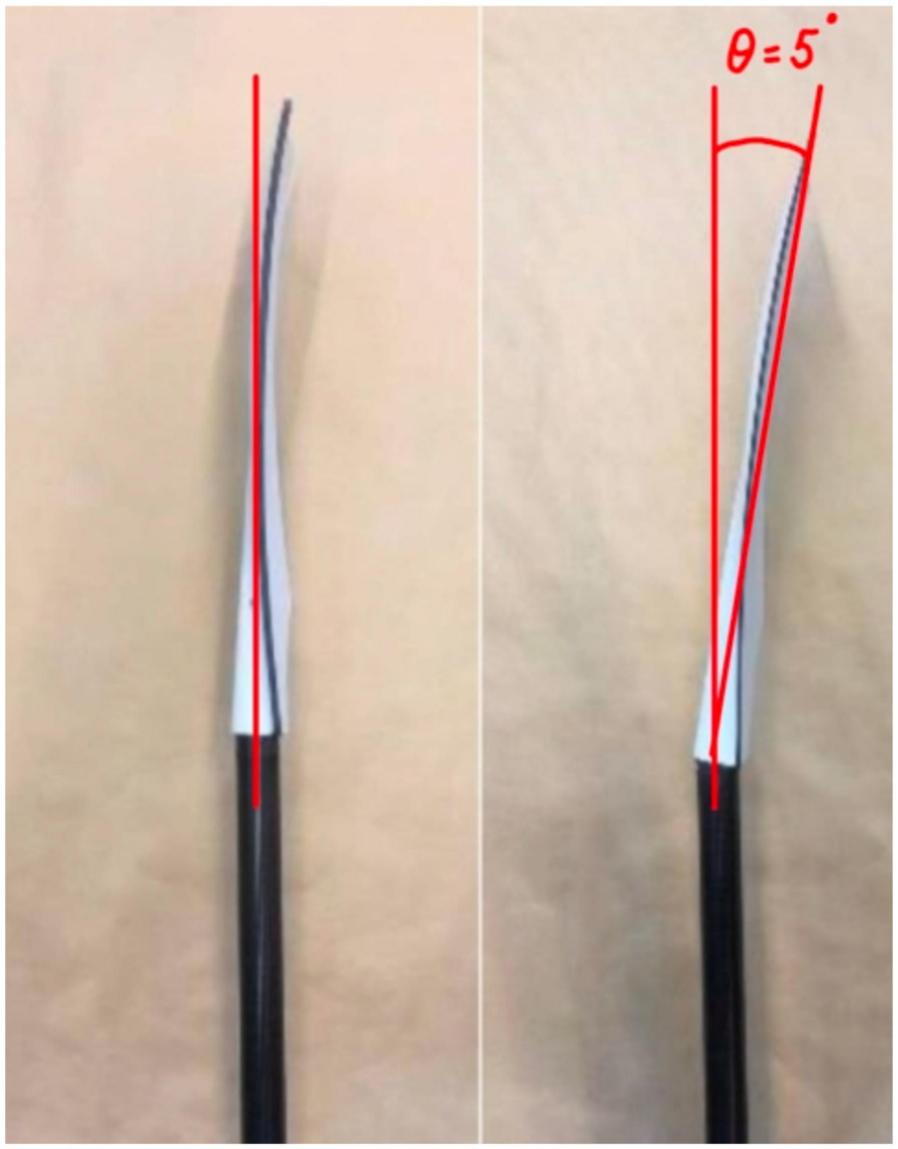
The traditional 0-degrees (left) and the 5-degrees oar blade (right).

The influence of oar blade angle on improved rowing efficiency has been shown in the fluid mechanics lab. It has been shown that altering the position and angle of the oar blade can significantly impact rowing effectiveness (2). In addition, positioning the rowing blade at a forward angle of 5–15 degrees relative to the oar's shaft may enhance rowing performance (1) (Figure 1), due to increased propulsive efficiency. When rowing at the same stroke rate, the blade's surface area can be increased by 4%–6% without increased effort of the athlete, leading to a boat speed increase of approximately 0.4% (1).

In rowing many innovations are focussed on performance indicators, while the influence on health is often neglected. Low back pain injuries are a common issue faced by rowers, impacting their performance, training, and overall quality of life (3). The incidence of low back pain in rowers ranged from 31.8% to 51% (4). One of the injury mechanisms from biomechanical perspective are the magnitude and number of loads on the lumbar spine joint, due to spinal segment (micro-) factures (5, 6). While rowing the lumbar spine is at maximal flexion at the catch and extends during the drive phase (7, 8). The drive phase the highest loads are reported on the lumbar spine (9), while the lumbar spine is extending. The erector spinae muscles extend the lumbar spine; high muscle activities are found during the driving phase. Several studies showed a positive relationship between the lumbar spine load and the level of muscle activity in lifting tasks (10, 11). In addition, muscle activity of the erector spinae is altered in rowers with a history of low back pain (7, 12). It is, therefore, interesting to investigate if alterations occur in muscle activity of the erector spinae, while rowing with an adjusted blade angle. It is hypothesized that an increase of muscle activity increases the chance of sustaining low back pain injuries, while a decrease could decrease injury risk (12, 13).

The change in blade angle of an oar may decrease the effort put forth while rowing (1, 14), it is unknown how this influences muscle activity on the rower. The aim of this study is to investigate the effect of an adjusted oar blade angle on the muscle activation of the erector spinae muscle in comparison to a normal oar blade angle.

## Method

### Participants

Seven experienced collegiate rowers (5 males, 2 females; M = 22.9 years, SD = 2.5; age range: 19–28) from a collegiate rowing club voluntarily participated in this study. All participants had a minimum of 3 years of rowing experience (M = 5.3 years, SD = 1.9), ensuring their familiarity with proper rowing technique and minimizing the impact of skill acquisition on performance metrics. Participants with a history of spinal or back-related injuries within the three months preceding the study were excluded. Informed consent was obtained prior to participation, and the study protocol was approved by the Human Research Ethics Committee (HREC) of TU Delft, approval number 4309.

### Procedure

All measurements were conducted on an open water canal. Prior to the rowing trials, participants performed maximum voluntary contractions (MVCs) to normalize muscle activity data. The MVC was obtained with the participant lying on their belly and being instructed to make a maximal extension, while two researchers pushed the the shoulder blades and ankles of the participant towards the ground. The activity was recorded using surface electromyography (EMG). Participants were instructed to gradually increase force to maximum effort over 3 s, maintain this contraction for 3 s, and then release over 3 s. Each MVC trial was repeated two to three times with 30 s of rest between trials. Subsequently, participants completed a warm-up consisting of 1,000 to 1,500 meters of rowing at moderate intensity. The rowers determined when they were ready to begin each trial. Then they performed two 500-meter rowing conditions in a single-person scull at approximately 80% of their maximal effort: one condition using traditional oars with a 0° blade angle and one condition using the oars with a 5° blade angle (Figure 1). A rest interval of at least 5 min was provided between the two conditions, and the order of the trials was randomized between participants to reduce the influence of fatigue.

### Data acquisition

Bipolar surface electromyography (EMG) was used to record muscle activity of the longissimus of the erector spinae (ES) bilaterally at the thoracic and lumbar level. A reference electrode was placed on the clavicula. Electrode placement followed the SENIAM guidelines (15). Specifically, electrodes were placed at the T10 vertebral level (thoracic ES) and L3 vertebral level (lumbar ES), with symmetrical positioning one or two finger widths lateral to the spinous process. The skin was shaved and cleaned with alcohol. Subsequently, disposable, pre-gelled Ag/AgCl surface electrodes (Blue Sensor N-00-S, Ambu Inc., USA) were applied with an inter-electrode distance of 20 mm and a gel contact area of 1 cm^2^ per electrode. All electrode cables were fixated to the skin using adhesive tape (Fixomull stretch) to minimize motion artifacts and prevent electrode detachment during dynamic rowing activity. Kinematics of the spine were measured using an electronic goniometer (Biometrics Ltd, UK). The goniometer was positioned between the L1 and L4 vertebrae to measure the lumbar spine flexion-extension. The goniometer provided a measurement range of ±150° and a 16-bit analog resolution. EMG signals were collected using a BioPlux research device (Plux biosignals, Arruda dos Vinhos, Portugal) with 16-bit analog resolution, a fixed gain of 506, and an analog band-pass filter between 25 and 500 Hz. The data were sampled at 2,000 Hz and stored locally on the device. EMG and goniometer data were recorded continuously throughout both 500 m rowing efforts.

### Data analysis

Four raw EMG signals from the thoracic and lumbar erector spinae were converted to voltage, rectified, and low-pass filtered (4th-order Butterworth, 40 Hz cutoff), using zero-phase filtering to obtain smoothed envelopes. The MVC trials were visually selected and over these trials the mean was calculated, for each muscle. All EMG recordings during rowing trials were divided by the MVC value and expressed as a percentage of the MVC. Kinematic data from the lumbar goniometer (angle between L1 and L4) were converted from raw ADC units to degrees and smoothed using a Savitzky-Golay filter (2nd order, 500-sample window). Rowing strokes were identified by detecting peak-to-peak patterns in the smoothed back angle signal. The peak that was used for the peak-to-peak pattern was the maximal lumbar extension angle. The moment of the “catch” was defined using the goniometer data of the lumbar spine. Specifically, it corresponded to the point of maximal lumbar flexion within each rowing cycle (16), as derived from the smoothed back angle signal. All selected strokes were visually checked. All EMG and goniometer data analyses were performed in Python (version 3.7, Python Software Foundation, https://www.python.org/).

The EMG signals were time-normalized to 101 samples (0%–100% of stroke duration) using linear interpolation, for each stroke. A stroke cycle normalization was defined from maximal lumbar extension till the next maximal lumbar extension, based on the goniometer data. Resampled stroke data were stored for four signals: thoracic and lumbar erector spinae (left and right). Resampled stroke snippets were averaged per participant and per oar condition (0° vs. 5° blade angle). For each signal, the mean and standard deviation were computed at each normalized time point. EMG signals were visualized using overlaid plots, allowing direct comparison between muscle activation.

### Statistics

The average data of the 70–120-time normalized stroke cycles for each participant were used for statistical analysis. To evaluate the statistical differences between rowing with a 0-degrees and a 5-degrees oar blade a paired t-test using 1-dimensional Statistical Parametric Mapping (SPM1D) was performed for the four EMG signals, using the SPM1D packages (17). This statistical method was used over traditional paired samples t-tests as it enables statistical analysis of an entire time series waveform. All statistical analyses were performed in Python (version 3.7, Python Software Foundation, https://www.python.org/). The level of statistical significance (α) was 0.05.

## Results

**Figure 2:**
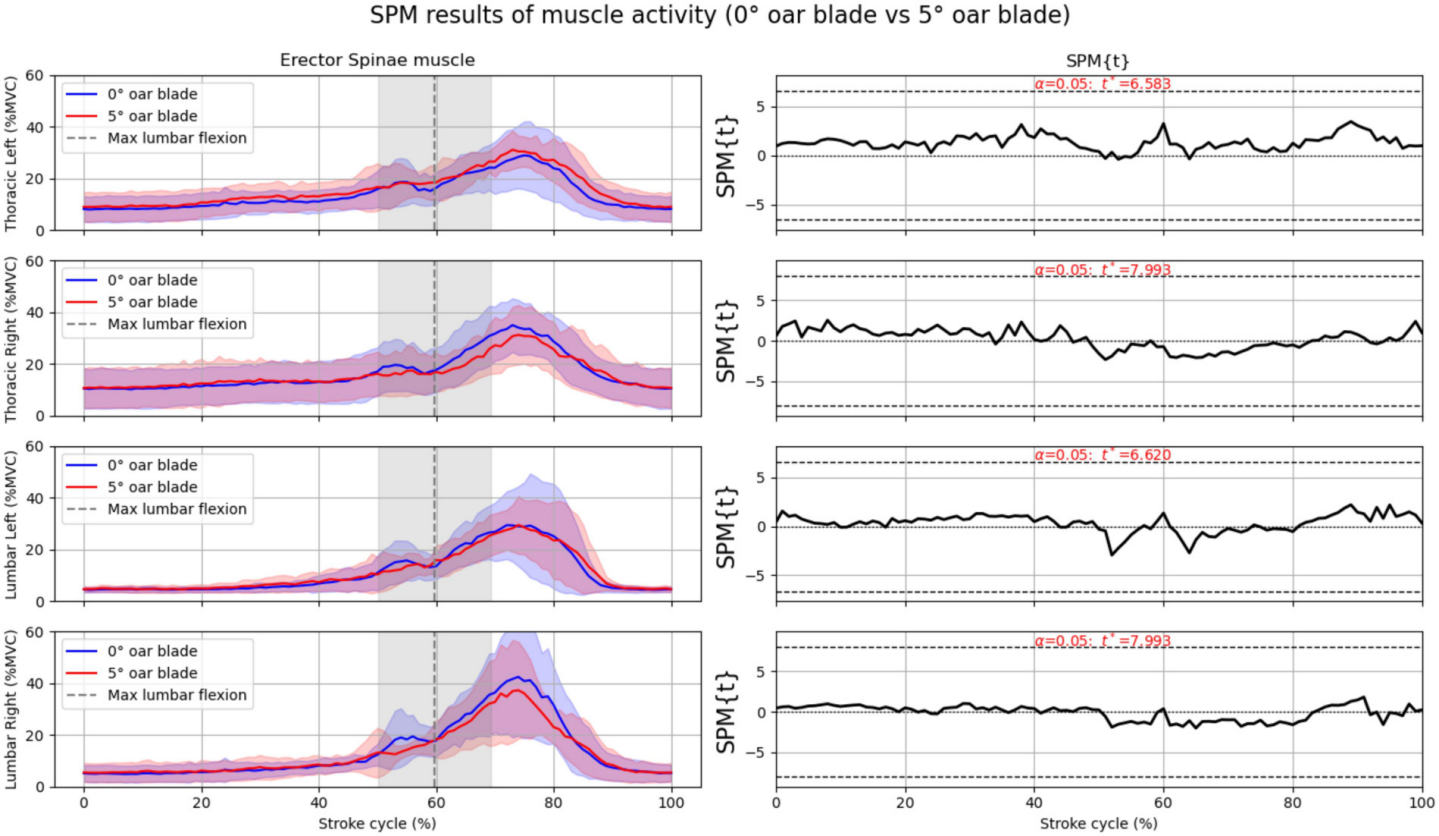
The left panels show the group level normalized muscle activation of the erector spinae muscle at four different locations. The thick line shows the mean over all 7 participants, and the standard deviation is shown as transparent area around the mean. The blue line is the muscle activity by the 0-degrees blade, and the red line is the muscle activation by the 5-degrees blade. The vertical dashed grey line represents the moment of maximal lumbar flexion. The shaded area around the lines is 1 standard deviation. The right panel show the SPM results between the 0-degrees blade and the 5-degrees blade. Be aware: the normalized activity ranges from 0% till 100%, here the *y*-axis scale ranges from 0% to 60% for better visualization.

In total 1,443, strokes were analyzed. After visually analyzing signals for artifacts, for instance due to loosening of electrodes, the thoracic right signal of participant 6 for both conditions and lumbar right for participant 7 in the zero condition (Figure 2) were excluded from the SPM analysis.

### Thoracic erector spinae

The mean maximum activation of the thoracic left erector spinae was 32.6% (SD = 11.7) with the 0° blade and 35.0% (SD = 5.3) with the 5° blade. For the thoracic right, activation was slightly higher with the 0° blade at 38.0% (SD = 8.1) compared to 35.6% (SD = 10.1) with the 5° blade. The SPM revealed no significant differences in muscle activity between the two oar blade conditions during a stroke cycle (Figure 2, right panel).

### Lumbar erector spinae

In the lumbar left, maximum activation was 36.7% (SD = 16.0) for the 0° blade and 35.6% (SD = 9.8) for the 5° blade. The lumbar right showed the largest difference in mean values, with 46.3% (SD = 20.8) for the 0° blade and 41.9% (SD = 17.4) for the 5° blade. The SPM revealed no significant differences in muscle activity between the two oar blade conditions (Figure 2, right panel).

### Rowing times

Table 1 shows the times performed over each 500 meters in both conditions for each participant. A paired sample t-test did not show significant differences between the times of both conditions, t(6) = 1.70, *p* = 0.14.

**Table 1:** Presents the rowing times over 500 meters rowing for each participant during each condition in seconds.

Participant	0 degrees blade (s)	5 degrees blade (s)
Participant 1	138	142
Participant 2	141	145
Participant 3	125	125
Participant 4	145	153
Participant 5	141	140
Participant 6	150	163
Participant 7	126	123
Average	138 (SD 9)	141 (SD 14)

## Discussion

The aim of this study was to investigate whether a 5-degrees oar blade angle alters the muscle activation of the erector spinae muscle in comparison to a normal oar blade angle during rowing. Muscle activity of the erector spinae did not show significant differences between rowing with a 5-degrees blade and a 0-degrees blade.

On-water rowing studies are limited, a systematic review of Legge et al. (18) reported only 27 biomechanical on water studies (18), none of the included studies investigated the biomechanics of the athlete. The reason of the limited studies is because of the limited instrumentations systems and the additional difficulty of variable environmental conditions (18), especially for athlete kinetics. The only way to investigate the effect of rowing blades on the lower back is to measure on-water. Studies based on lifting activities show a relationship between EMG and the extensor moment of the lower back, which is a good proxy for the low back load (19, 20). Higher muscle activity results in higher extensor moment and thus low back load. Therefore, we investigated the EMG of the erector spinae muscles as indicator of low back load during on-water rowing.

To our knowledge this is the first study that measured muscle activity of the erector spinae during on water rowing. On group level, maximal muscle activities were found between 32% and 46% in our study. The lumbar muscle activities were higher compared to the thoracic muscle activities on both sides. Maximal muscle activities were reported after maximal lumbar flexion; indicated as the moment of the “catch” during rowing (16). The muscle activation patterns over a stroke cycle are comparable with studies that measured the muscle activity on a rowing ergometer (7, 12, 21). However, the peak values were slightly lower in our study, as Yamashita and Caldwell reported muscle activities of 50% in healthy rowers. This could be explained by the differences between rowing on-water and on an ergometer. In contrast, Flemming et al. (22) found higher muscle activities in the lower legs during on-water rowing (m. rectus femoris and m. vastus lateralis) compared to ergometer rowing (22). The differences in muscle activation could also be explained by the differences in methodology. In the ergometer studies participants were instructed to row at maximal effort for 500 m and 2000m, while in our study they were instructed to row 500 m at 80% of their maximal effort.

**Figure 3:**
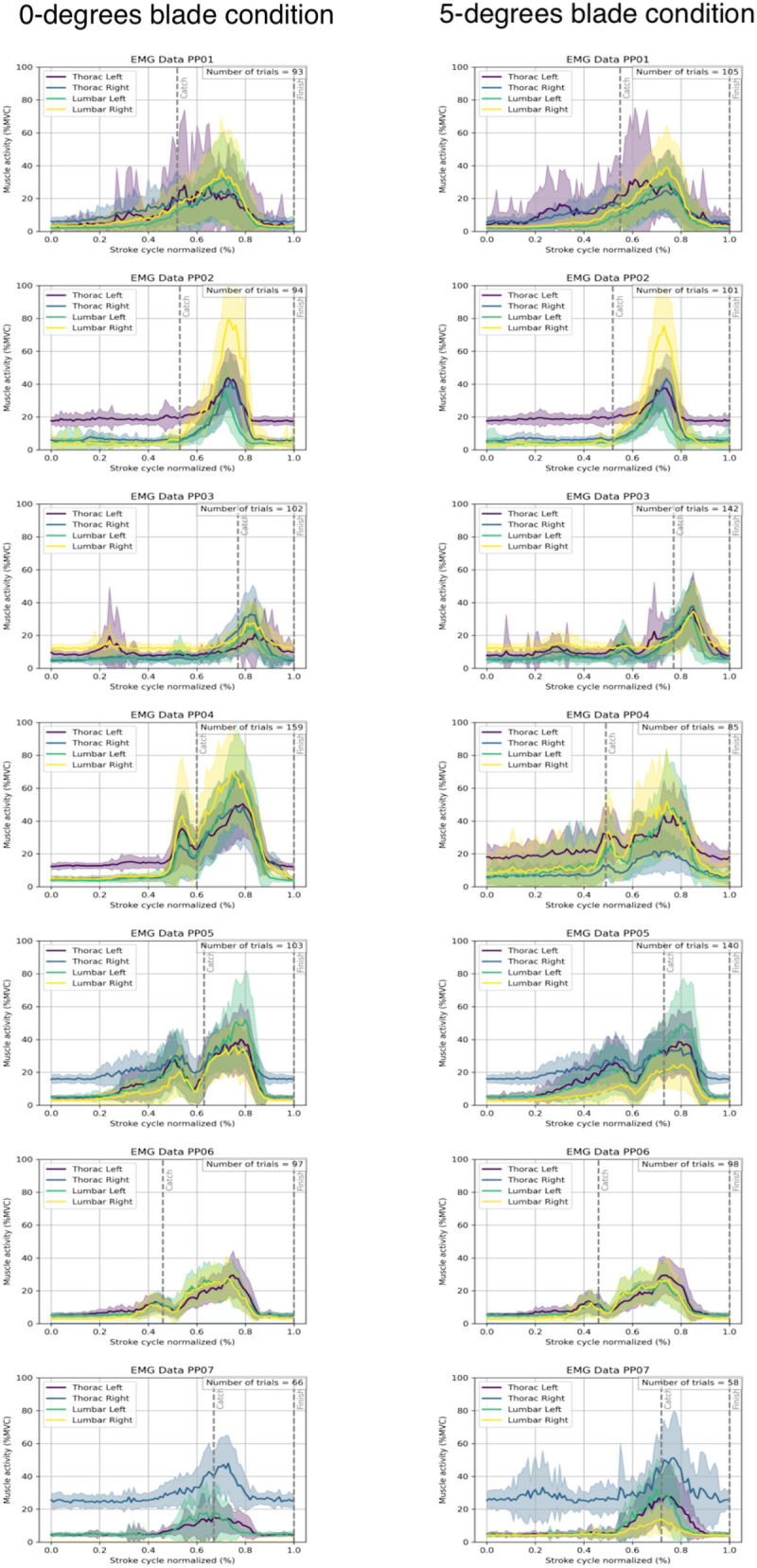
The muscle activities of each participant at four muscle locations of the erector spinae longissimus muscle. The left panels are the muscle activity of the 0-degrees oar blade and the right panels of the 5-degrees oar blade. The dashed lines represents the catch and the finish of the stroke. Be aware: the number of strokes, are the number of strokes analyzed, not the number of strokes that the participant made.

In our study and in other EMG rowing studies, analyses are performed on group level. To our knowledge no other study investigated the within-individual muscle activity. While analyzing the EMG of each participant separately, we remarked two differences in muscle activity during rowing. First, we found differences between muscle activity patterns. All participants showed the highest muscle activity after the moment of the catch; however, some participants (participant 4,5 and 6) showed a second peak just before the catch. Second, there is a large difference in maximal activity, participant 2 and 4 show values of 79%, whereas participants 3 and 6 show maximal muscle activities of only 30% (Figure 3, yellow line). If higher muscle activity of the erector spinae is related to LBP this should be investigated in future studies. Yamashita et al. (12) found that rowers with LBP showed higher muscle activity of the thoracic erector spinae. The question that remains is whether this is a result of the LBP or that it is a biomarker for low back pain. Future longitudinal studies should investigate how and if muscle activity is related to LBP injuries.

This study contains several limitations. Conducting the measurements on water increases ecological validity but also introduces external factors such as wind and waves, which may have influenced muscle activation. An increase head wind between conditions could influence the rowers need to apply more power to maintain comparable speeds, which may have affected muscle activation. However, Table 1 shows that speeds were not different between the two conditions, nevertheless power output was not measured and therefore not controlled. External factors could also be one of the explanations of the differences between individual EMG patterns (Figure 3). The number of analyzed strokes was not exactly equal across oar conditions, as strokes with poor EMG quality (e.g., due to loose electrodes) were excluded to ensure reliable data. However, this minor variation is unlikely to have influenced the results, since condition comparisons in the SPM analysis were based on the mean of all valid strokes per participant. Another limitation is the relatively small number of participants (*N* = 7). A limited sample size restricts the generalizability of the findings. However, this was partly counterbalanced by the large number of analyzed strokes per condition. For each participant, at least 70 consecutive strokes were included, resulting in a more accurate dataset compared to earlier studies that examined only three to five strokes per condition (7, 12).

## Conclusion

New innovations in sport equipment are most of the time focused on enhancing performance, while the effect on injury risk is not investigated. This study investigated the influence of oar blade angle on low back muscle activity. No significant differences in muscle activation patterns were found between the 0 ° and 5 ° oar blade conditions during on-water rowing. These prelimary findings suggest that oar blade angle adjustments might not increase erector spinae activations, although further research with larger cohorts is needed to confirm this observation.

## Data Availability

The datasets presented in this study can be found in online repositories. The names of the repository/repositories and accession number(s) can be found below: 10.4121/cee23508-729f-4355-9a48-81e41d312000.

## References

[1] van Nieuwburg W van Spreuwel BJJ Tran MTK Yang MD Greidanus A Mulder G Improving rowing performance by adjusting oar blade size and angle. Front Sports Act Living. (2023) 5:1109494. 10.3389/fspor.2023.1109494 36969962 PMC10034370

[2] Grift EJ. The hydrodynamics of rowing propulsion: an experimental study. (2020). 10.4233/uuid:defe4405-a1c1-4e18-af48-7a93fcd55152

[3] Wilson F Thornton JS Wilkie K Hartvigsen J Vinther A Ackerman KE 2021 Consensus statement for preventing and managing low back pain in elite and subelite adult rowers. Br J Sports Med. (2021) 55:893–9. 10.1136/bjsports-2020-103385 33685861

[4] Wilson F Gissane C McGregor A. Ergometer training volume and previous injury predict back pain in rowing; strategies for injury prevention and rehabilitation. Br J Sports Med. (2014) 48:1534–7. 10.1136/bjsports-2014-093968 25257230

[5] Reid DA Mcnair PJ. Factors contributing to low back pain in rowers. Br J Sports Med. (2000) 34:321–2. 10.1136/bjsm.34.5.321 11049136 PMC1756238

[6] Coenen P Kingma I Boot CRL Bongers PM van Dieën JH. The contribution of load magnitude and number of load cycles to cumulative low-back load estimations: a study based on *in-vitro* compression data. Clin Biomech. (2012) 27:1083–6. 10.1016/j.clinbiomech.2012.07.010 22877832

[7] Caldwell JS McNair PJ Williams M. The effects of repetitive motion on lumbar flexion and erector spinae muscle activity in rowers. Clin Biomech. (2003) 18:704–11. 10.1016/S0268-0033(03)00117-7 12957556

[8] Wilson F Gissane C Gormley J Simms C. Sagittal plane motion of the lumbar spine during ergometer and single scull rowing. Sports Biomech. (2013) 12:132–42. 10.1080/14763141.2012.726640 23898686

[9] Buckeridge EM Bull AMJ McGregor AH. Incremental training intensities increases loads on the lower back of elite female rowers. J Sports Sci. (2016) 34:369–78. 10.1080/02640414.2015.1056821 26090702

[10] Potvin JR Norman RW McGill SM. Mechanically corrected EMG for the continuous estimation of erector spinae muscle loading during repetitive lifting. Eur J Appl Physiol Occup Physiol. (1996) 74:119–32. 10.1007/BF00376504 8891510

[11] Callaghan JP Gunning JL McGill SM. The relationship between lumbar spine load and muscle activity during extensor exercises. Phys Ther. (1998) 78:8–18. 10.1093/ptj/78.1.8 9442191

[12] Yamashita M Ishida T Osuka S Watanabe K Samukawa M Kasahara S Trunk muscle activities during ergometer rowing in rowers with and without low back pain. J Sports Sci Med. (2023) 22:338. 10.52082/jssm.2023.338 37293422 PMC10245001

[13] Martinez-Valdes E Wilson F Fleming N McDonnell S-J Horgan A Falla D. Rowers with a recent history of low back pain engage different regions of the lumbar erector spinae during rowing. J Sci Med Sport. (2019) 22:1206–12. 10.1016/j.jsams.2019.07.007 31371258

[14] Grift EJ Tummers MJ Westerweel J. Hydrodynamics of rowing propulsion. J Fluid Mech. (2021) 918:A29. 10.1017/jfm.2021.318

[15] Hermens HJ Freriks B Merletti R Stegeman D Blok J Rau G European Recommendations for surface electromyography. Roessingh Res Dev. (1999) 8:13–54.

[16] Sekine C Matsunaga N Kaneoka K. Changes in lumbopelvic motion and trunk muscle activity during 2000m rowing ergometer trial. Int J Sport Health Sci. (2021) 19:47–57. 10.5432/ijshs.202048

[17] Pataky TC. One-dimensional statistical parametric mapping in python. Comput Methods Biomech Biomed Engin. (2012) 15:295–301. 10.1080/10255842.2010.527837 21756121

[18] Legge N Draper C Slattery K O’Meara D Watsford M. On-water rowing biomechanical assessment: a systematic scoping review. Sports Med Open. (2024) 10:101. 10.1186/s40798-024-00760-2 39331267 PMC11436553

[19] Seroussi RE Pope MH. The relationship between trunk muscle electromyography and lifting moments in the sagittal and frontal planes. J Biomech. (1987) 20:135–46. 10.1016/0021-9290(87)90305-8 3571294

[20] Mouton LJ Hof AL de Jongh HJ Eisma WH. Influence of posture on the relation between surface electromyogram amplitude and back muscle moment: consequences for the use of surface electromyogram to measure back load. Clin Biomech. (1991) 6:245–51. 10.1016/0268-0033(91)90053-S 23915570

[21] Pollock CL Jenkyn TR Jones IANC Ivanova TD Garland SJ. Electromyography and kinematics of the trunk during rowing in elite female rowers. Med Sci Sports Exerc. (2009) 41:628–36. 10.1249/MSS.0b013e31818c1300 19204587

[22] Fleming N Donne B Mahony N. A comparison of electromyography and stroke kinematics during ergometer and on-water rowing. J Sports Sci. (2014) 32:1127–38. 10.1080/02640414.2014.886128 24576175

